# Condition of the Patients

**Published:** 1921-08-20

**Authors:** 


### THE PROBLEM OF THE INSANE.

### II. Condition of the Patients.

The accusations which Dr. Lomax and others bring against asylum administration may doubtless in some instances be justified. It would be an amazing thing if no examples of ill-treatment, of innutritious and badly-cooked food, of too long seclusion in side-rooms, and so on, occurred. If anyone were to say that everything is for the best and nothing needs criticism in such a vast system, we might well suspect either the adequacy of his knowledge or his *bona fides*. It is quite another thing to maintain that the whole system is effete, that the conduct of the great majority of the staff is flagitious, and that nothing is done to bring about a cure in the recoverable cases. As a matter of fact, in no country are the insane generally so well tended and so well housed as in our own.

It is nonsense—and pernicious nonsense because of the depressing effect it has on anxious relatives and friends—to say that no attempt is made to cure patients. Medical records show that mental patients are being treated and are recovering daily in asylums throughout the country, to be discharged in due course to the care of their friends and to return to their avocations. Anyone who has practical knowledge of these matters knows that this is so—but they also know full well with what poor material those have to deal who are endeavouring to rehabilitate the mentally disordered. If they are pessimistic as to the possibility of bringing about cures in a large proportion of the cases, it is because they know too much to share the optimism of the ignorant.

It is difficult to make those who have no practical knowledge of the insane understand the problems which have to be faced in dealing with many of the cases. To the casual observer it seems dreadful to see a patient in a small, darkened room—designated as a "cell" by those who write for effect—lying on a mattress on the floor; or to find patients sitting or standing for long periods in one position; or to find them at a given moment dirty and untidy. Yet many people who have passed through such phases are out and about in the world, sane and competent to carry on their work. It must be realised that patients are noisy, violent, destructive, filthy in their habits, obscene in speech, not because they are in asylums but because they are insane. They become like this before they ever reach the asylum—as their neighbours know to their cost—if there is undue delay in placing them under care. Nor does anyone who has a conception of what is taking place in the nervous system wonder at their conduct. As Hughlings Jackson has pointed out, there is in nervous disorder an over-action of the part not already involved—that is, there is a lack of inhibition. Self-restraint and orderly social conduct are acquired late in evolution, and, therefore, they soon give way where there is disease or disorder; and the person reverts to a more primitive type of behaviour—that of the young child, or even like that of an animal. Frequently it is not possible to nurse such patients in an ordinary bedroom or in a dormitory. To do so it would be necessary either to struggle continuously with them or to drug them to a state of unconsciousness. There are dangers attaching to both courses. If the first is adopted the patient may injure himself or the nurses—and, in any case, he is exhausting his remaining strength. It is hardly necessary to dwell upon the risks of intensive drugging. It is not feasible to place such a patient in a room with a bedstead and furniture because he may—and often does—destroy what he can and use the parts of the bedstead to batter the walls and the door. On the other hand, a patient who has been struggling violently will settle down when placed in a room by himself, especially if the room is darkened. We do not expose an inflamed eye to a bright light: surely it is not illogical to deprive an irritable and excitable brain of all stimuli which may tend to increase the disorder. There is nothing inhumane in secluding such patients; indeed, there is much more inhumanity in fighting and struggling with them. What other means we are to adopt—except mechanical restraint, which must be resorted to in, for example, surgical cases, but the routine use of which is to be deprecated—it is difficult to see. Nor is this a question which can be argued on theoretic lines: it is a matter of experience. It is in the abuse of seclusion that it becomes an evil thing; but the alleged abuse seems to be much exaggerated. Without a judicious resort to seclusion a ward may be rendered a pandemonium by the presence in it of even one or two restless, noisy, violent patients. And it has yet to be proved that seclusion is harmful to the patients who undergo it: rather, as already stated, it can be shown to be actually beneficial. It should, of course, be clearly understood that all such rooms must be properly heated; and the Board of Control should have full power to dictate to the governing body of any asylum that such heating is imperative.

(*To be continued.*)

* The first article appeared August 13, p. 329.

